# A previously unreported *ELOVL4* frameshift variant in a patient with early severe cognitive decline, parkinsonism, and cerebellar ataxia: A case report

**DOI:** 10.1097/MD.0000000000050787

**Published:** 2026-09-25

**Authors:** Jingtong Zhou, Xiaohui Wen, Zhijun Lin

**Affiliations:** a Department of Neurology, Affiliated Hospital of Guangdong Medical University, Zhanjiang, Guangdong, China.

**Keywords:** case report, *ELOVL4*, frameshift variant, parkinsonism, spinocerebellar ataxia type 34

## Abstract

**Rationale:**

Spinocerebellar ataxia type 34 is a rare autosomal dominant ataxia associated with heterozygous elongation of very long-chain fatty acids protein 4 (*ELOVL4*) variants. Parkinsonism and severe early cognitive impairment are uncommon, and frameshift variants remain poorly characterized.

**Patient concerns:**

A 35-year-old man presented with a 5-year history of progressive cognitive decline, bradykinesia, resting tremor, dysarthria, gait instability, and severe constipation.

**Diagnoses:**

Examination showed bilateral resting tremor, rigidity, limb and gait ataxia, and hyperreflexia. The Movement Disorder Society–Unified Parkinson’s Disease Rating Scale Part III score was 60, and the Scale for the Assessment and Rating of Ataxia score was 21. Cognitive testing showed severe impairment (Mini-Mental State Examination, 10; Montreal Cognitive Assessment, 8). Brain magnetic resonance imaging demonstrated marked cerebellar and generalized cerebral atrophy, and susceptibility-weighted imaging showed reduced dorsolateral nigral hyperintensity. Whole-exome sequencing identified a previously unreported heterozygous *ELOVL4* frameshift variant, NM_022726.4:c.766_767delinsG (p.Ile256AlafsTer28). The clinical genetic testing report classified the variant as a variant of uncertain significance. Its predicted truncating effect and the patient’s phenotypic overlap with *ELOVL4*-related ataxia supported its consideration as a potentially clinically relevant candidate variant. However, Sanger confirmation and family segregation analysis were unavailable.

**Interventions:**

The patient received eperisone hydrochloride, levodopa/benserazide, piribedil prolonged-release tablets, adenosyl cobalamin, and vitamin B1.

**Outcomes:**

During 1 year of telephone and video follow-up, he reported mild improvement in tremor, gait difficulty, and rigidity, whereas dysarthria, slowing of eye movements, and constipation worsened. Repeat standardized assessments were unavailable.

**Lessons:**

This case describes a previously unreported *ELOVL4* frameshift variant in a patient with cerebellar ataxia, parkinsonism, severe cognitive impairment, and autonomic dysfunction. Genetic testing may facilitate differentiation of hereditary ataxia from atypical degenerative syndromes. Additional molecular validation, segregation analysis, and functional studies are required to establish pathogenicity and genotype–phenotype relationships.

## 1. Introduction

Spinocerebellar ataxia type 34 (SCA34) is a rare autosomal dominant cerebellar ataxia associated with heterozygous variants in elongation of very long-chain fatty acids protein 4 (*ELOVL4*). In a Japanese ataxia cohort, SCA34 accounted for approximately 0.2% of patients with ataxia.^[1]^ *ELOVL4* encodes a fatty acid elongase required for the biosynthesis of very-long-chain saturated and polyunsaturated fatty acids, which are important for normal function in the brain, retina, and skin.^[2,3]^

Importantly, *ELOVL4*-related disorders comprise several clinically and genetically distinct entities. Heterozygous variants have been associated with autosomal dominant SCA34 and autosomal dominant Stargardt-like macular dystrophy, whereas biallelic variants cause a severe neuro-ichthyotic syndrome characterized by ichthyosis, developmental impairment, epilepsy, and spasticity.^[2,4–8]^ These disorders differ in their inheritance patterns, predominant organ involvement, and clinical course and should therefore not be regarded as a single continuous phenotypic spectrum.

In the present case, we identified a previously unreported frameshift variant in *ELOVL4* (NM_022726.4:c.766_767delinsG). To the best of our knowledge, this is the first frameshift variant reported in a patient with an SCA34-like phenotype. The variant is predicted to introduce a premature termination codon and result in a truncated *ELOVL4* protein. However, its molecular consequences and contribution to the patient’s phenotype remain uncertain. This case may broaden the clinical spectrum associated with *ELOVL4*-related ataxia and highlights the need for additional cases, segregation analyses, and functional studies to clarify its pathogenic significance and genotype–phenotype correlations.

## 2. Ethics approval and consent

According to the policy of the Ethics Committee of the Affiliated Hospital of Guangdong Medical University, formal ethical review was not required for this single-patient case report because it did not constitute interventional or systematic human-subject research. Written informed consent was obtained from the patient for publication of his clinical information, genetic findings, and deidentified clinical images. The consent process was conducted in Chinese using language appropriate to the patient’s level of understanding.

## 3. Case presentation

Patient: Male, 35 years old at presentation. Education level: Primary school.

History of presenting complaint: The patient had progressive cognitive decline and bradykinesia for approximately 5 years. His initial symptoms included slowed responsiveness, impaired concentration and memory, and easy fatigability. Parkinsonian features subsequently developed, including resting tremor that began in the right upper limb and later involved the left upper limb and neck, as well as increased muscle tone. He also had sleep disturbances, including vivid dreams and sleep talking, and his speech became progressively slurred. Autonomic features included significant constipation, with bowel movements every 4 to 7 days.

Examination: Neurologic examination revealed resting tremor in both upper limbs, lower-limb ataxia with a wide-based gait, action-related ataxia in the left upper limb, generalized increased muscle tone, and hyperreflexia. The Babinski sign was negative. No obvious nystagmus or slowed saccades were documented.

Neuropsychological testing: Mini-Mental State Examination = 10; Montreal Cognitive Assessment (MoCA) = 8 (education level: primary school). Elevated anxiety and depression scores were also noted.

Medical, family, and psychosocial history: No similar neurologic illness was reported in first-degree relatives. His psychosocial history was unremarkable, with no reported substance use, psychiatric illness, or other relevant factors.

## 4. Investigations

Laboratory tests: Complete blood count, infection markers, and liver and renal function tests were within normal limits. Ceruloplasmin was 18.5 mg/dL (reference range, 22–58 mg/dL). Ophthalmologic examination showed no Kayser–Fleischer rings.

Neuroimaging: Brain magnetic resonance imaging (MRI) showed diffuse cerebral atrophy and marked bilateral cerebellar hemispheric atrophy (Figs. 1 and 2). Susceptibility-weighted imaging (SWI) showed an indistinct right-sided “swallow-tail” sign (Fig. 2). Diffusion-weighted imaging showed no acute infarction. The hot cross bun sign (HCBS) typically seen in multiple system atrophy (MSA) was not identified.

**Figure 1.**
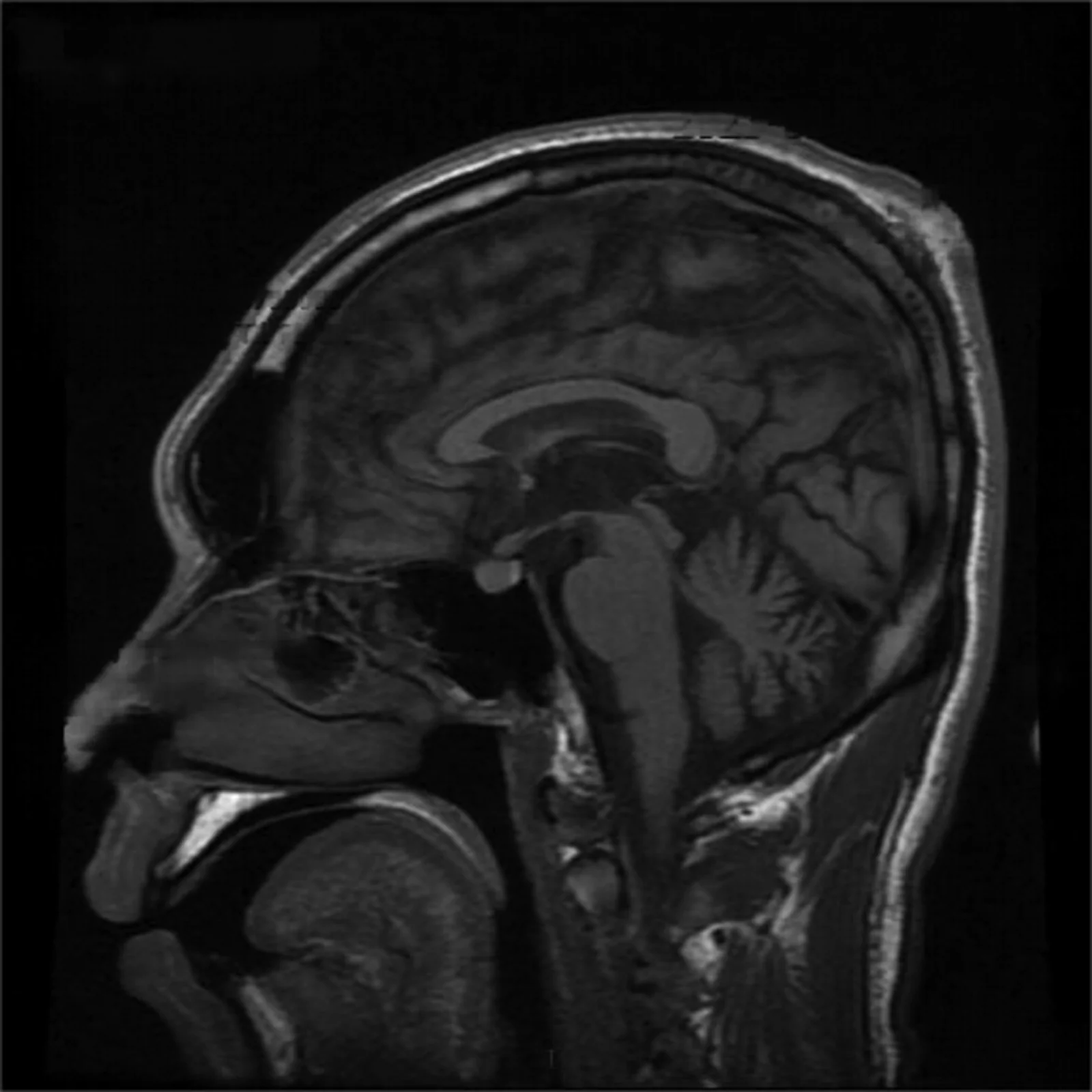
Diffuse cerebral atrophy and marked bilateral cerebellar hemispheric atrophy on a T1-weighted fluid-attenuated inversion recovery magnetic resonance image.

**Figure 2.**
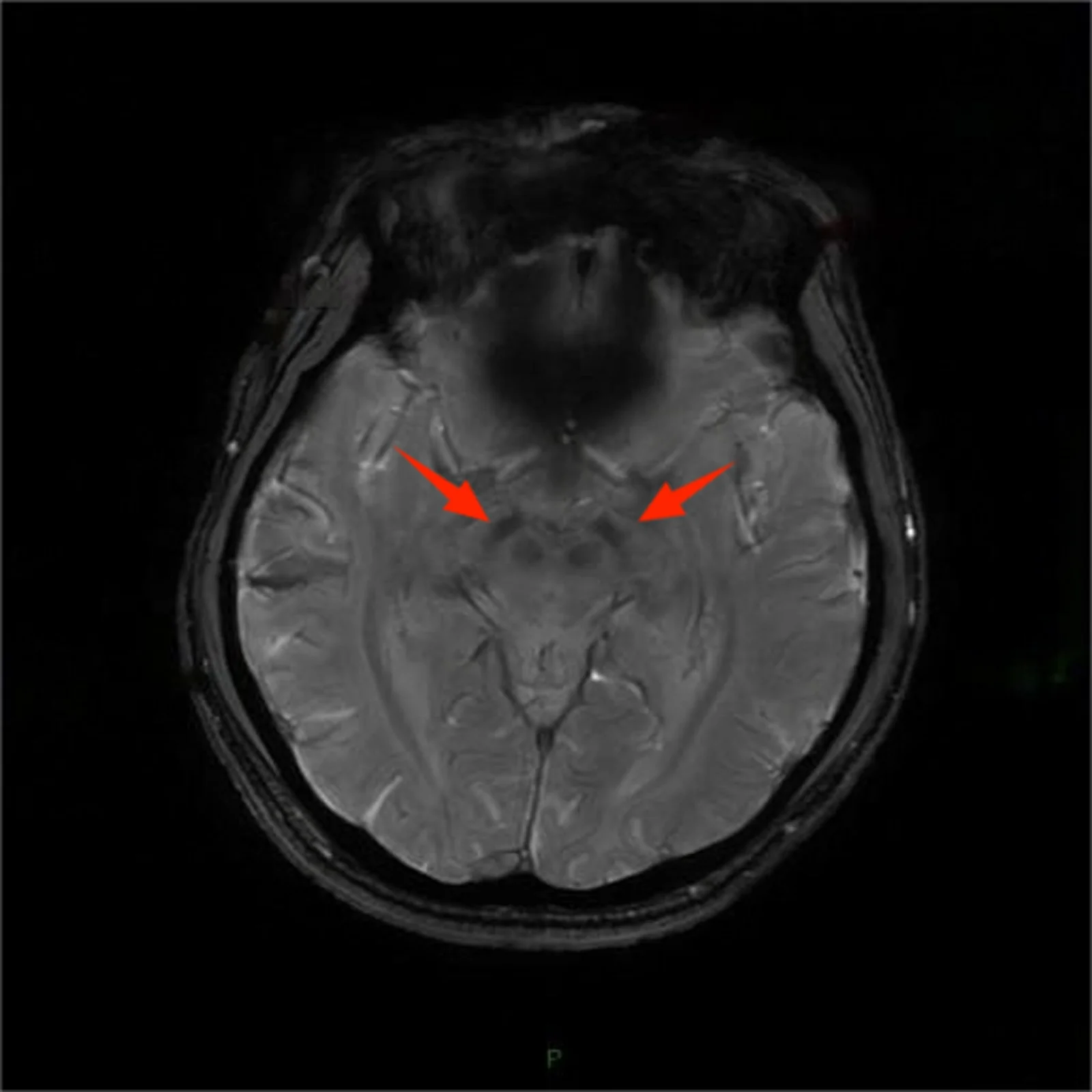
Reduced visibility of the dorsolateral nigral hyperintensity (“swallow-tail” sign) on susceptibility-weighted imaging, more pronounced on the right.

Genetic testing: Whole-exome sequencing identified a heterozygous *ELOVL4* variant, NM_022726.4:c.766_767delinsG, predicted to result in p.Ile256AlafsTer28 and premature truncation of the encoded protein. The clinical genetic testing report classified the variant as a variant of uncertain significance. Independent Sanger confirmation and family segregation analysis were unavailable.

## 5. Differential diagnosis

The patient’s clinical presentation, characterized by cognitive impairment, parkinsonian symptoms, and cerebellar ataxia, suggested a multisystem neurodegenerative process. Given the overlapping symptoms and their slow progression, the following differential diagnoses were prioritized.

### 5.1. Multiple system atrophy

#### 5.1.1. Supporting evidence

The presence of cerebellar ataxia, reflected by the wide-based gait, and autonomic dysfunction, such as constipation, is consistent with the MSA-C subtype.

#### 5.1.2. Contradictory evidence

Parkinsonian symptoms in MSA typically respond poorly to levodopa; however, in this case, the patient’s tremor and increased muscle tone partially improved with medication. Brain MRI did not reveal the classic HCBS seen in MSA-C. Although the HCBS can also be observed in SCA34, its presence or absence alone cannot reliably distinguish SCA34 from MSA-C. In MSA, dysarthria also progresses more rapidly, and most patients develop slurred speech within 2 to 3 years.

#### 5.1.3. Conclusion

MSA-C remained an important differential diagnosis. The absence of an HCBS and the reported partial symptomatic response to dopaminergic therapy did not independently exclude MSA. However, the patient’s young age, severe cognitive impairment, long disease course, and identification of a candidate hereditary ataxia variant favored consideration of a genetic disorder, while acknowledging that the causal significance of the variant remained uncertain.

### 5.2. Other hereditary ataxias (e.g., SCA5)

#### 5.2.1. Exclusion rationale

Among patients with SCA5, downbeat nystagmus is frequently one of the earliest signs and typically precedes other clinical symptoms, a feature rarely seen in other SCA subtypes. SCA5 generally does not present with marked cognitive impairment and seldom affects respiration, swallowing, or other bodily functions. As an autosomal dominant condition, similar cases are commonly observed in affected families. However, the present case appeared to be a solitary occurrence within the family, was characterized by progressive cognitive decline, and showed no obvious nystagmus on examination; therefore, SCA5 was temporarily excluded.

#### 5.2.2. Conclusion

No clearly diagnostic variant for another hereditary ataxia was identified by the available exome analysis; however, the testing performed did not exclude all repeat-expansion, structural, noncoding, or poorly covered variants.

### 5.3. Secondary causes (e.g., metabolic or toxic disorders)

Serum ceruloplasmin was mildly decreased at 18.5 mg/dL (laboratory reference range, 22–58 mg/dL), and no Kayser–Fleischer rings were identified. These findings did not confirm Wilson disease; however, Wilson disease could not be completely excluded because 24-hour urinary copper excretion, quantitative hepatic copper analysis, and comprehensive *ATP7B* testing were unavailable. There was no history of toxic exposure or drug-related toxicity, which argued against toxic encephalopathy.

### 5.4. Final diagnostic interpretation

The combination of progressive cerebellar ataxia, marked cerebellar atrophy, parkinsonism, severe cognitive impairment, autonomic dysfunction, and a previously unreported heterozygous *ELOVL4* frameshift variant supported a working diagnosis of an *ELOVL4*-related ataxia with an SCA34-like phenotype. The predicted truncating effect of the variant and its phenotypic concordance suggested potential clinical relevance. However, because the variant was not independently confirmed by Sanger sequencing or evaluated by family segregation and functional analyses, a definitive causal relationship could not be established.

### 5.5. Genetic finding

The *ELOVL4* c.766_767delinsG variant is predicted to cause a frameshift and premature protein truncation, resulting in p.Ile256AlafsTer28. However, it was classified as a variant of uncertain significance in the clinical genetic testing report. Sanger confirmation, family segregation analysis, and functional studies were unavailable.

### 5.6. Phenotypic compatibility

The patient’s combination of cerebellar ataxia, cerebellar atrophy, parkinsonism, cognitive impairment, and autonomic dysfunction is compatible with the broad clinical spectrum reported in *ELOVL4*-related ataxia. Nevertheless, phenotypic compatibility alone is insufficient to establish a causal relationship between the variant and the complete clinical presentation.

### 5.7. Consideration of alternative diagnoses

MSA of the cerebellar type, other hereditary ataxias, Wilson disease, and toxic or metabolic disorders were considered. These diagnoses were regarded as less likely based on the available clinical, laboratory, neuroimaging, and genetic findings, although they could not all be excluded with absolute certainty.

### 5.8. Diagnostic interpretation

Based on the overall clinical and genetic findings, the patient was considered to have an SCA34-like phenotype associated with a previously unreported *ELOVL4* frameshift variant. Additional molecular confirmation, family-based analysis, and functional studies are required before the variant can be definitively classified as disease-causing.

## 6. Treatment

During hospitalization, the patient had a Movement Disorder Society–Unified Parkinson’s Disease Rating Scale Part III (MDS-UPDRS Part III) score of 60 and a Scale for the Assessment and Rating of Ataxia (SARA) score of 21, indicating substantial parkinsonism and ataxia. Treatment consisted of eperisone hydrochloride 50 mg 3 times daily for increased muscle tone and muscle spasm, levodopa/benserazide 0.125 g twice daily, and piribedil prolonged-release tablets 50 mg once daily for parkinsonian symptoms. Adenosyl cobalamin and vitamin B1 were also administered as supportive therapy.

The patient reportedly continued dopaminergic treatment for several months after discharge. However, the exact duration of treatment, subsequent dose adjustments, medication adherence, and maintenance regimen could not be confirmed because he did not return to our hospital for further prescriptions or follow-up evaluation.

## 7. Outcome and follow-up

The patient was followed for 1 year, mainly by telephone and video consultation. Because of financial constraints and the long distance between his home and our hospital, he declined repeated recommendations to return for further treatment and in-person neurological assessment. As a result, repeat MDS-UPDRS Part III and SARA evaluations were not available.

During follow-up, the patient reported mild improvement in tremor, walking difficulty, and increased muscle tone after dopaminergic treatment. In contrast, dysarthria and slowing of eye movements gradually worsened, and constipation became more severe. These findings suggested a modest symptomatic benefit for some parkinsonian features, whereas the cerebellar, oculomotor, and autonomic manifestations continued to progress. No treatment-related adverse or unanticipated events were reported during the available follow-up period. The patient’s clinical course is summarized in Table 1.

**Table 1:** Timeline of the patient’s clinical course.

Time	Clinical course and main findings	Investigations, treatment, and outcomes
Childhood	According to the retrospective account provided by the patient and his family, he had difficulty maintaining attention, daytime drowsiness, and poorer academic and physical performance than his peers.	No formal neurological evaluation was documented during this period.
Approximately 5 yr before presentation	Progressive cognitive slowing, impaired concentration and memory, easy fatigability, and bradykinesia became apparent.	No definitive diagnosis was established despite previous medical evaluations.
During the subsequent disease course	Resting tremor initially developed in the right upper limb and later involved the left upper limb and neck. Increased muscle tone, dysarthria, gait instability, vivid dreams, sleep talking, and constipation gradually developed.	The patient underwent previous brain imaging at other hospitals, but no definitive diagnosis was made.
At presentation, age 35 yr	Neurological examination showed bilateral resting tremor, increased muscle tone, limb and gait ataxia, a wide-based gait, and hyperreflexia. Severe cognitive impairment was documented.	MDS-UPDRS Part III score: 60; SARA score: 21; MMSE score: 10; MoCA score: 8. Brain MRI showed diffuse cerebral and marked cerebellar atrophy. SWI showed reduced visibility of the dorsolateral nigral hyperintensity. Whole-exome sequencing identified the heterozygous *ELOVL4* c.766_767delinsG (p.Ile256AlafsTer28) variant.
During hospitalization	Parkinsonian, cerebellar, cognitive, and autonomic manifestations were assessed.	Eperisone hydrochloride 50 mg 3 times daily, levodopa/benserazide 0.125 g twice daily, piribedil prolonged-release tablets 50 mg once daily, adenosyl cobalamin, and vitamin B1 were administered.
First several months after discharge	The patient reportedly continued dopaminergic treatment.	The exact duration, dose adjustments, adherence, and maintenance regimen could not be verified because he did not return for further prescriptions or in-person assessment.
At 1-yr follow-up	The patient reported mild improvement in tremor, walking difficulty, and increased muscle tone. Dysarthria, slowing of eye movements, and constipation had worsened.	Follow-up was conducted mainly by telephone and video consultation. Repeat MDS-UPDRS Part III and SARA assessments were unavailable because the patient declined in-person reassessment owing to travel distance and financial constraints.

MDS-UPDRS = Movement Disorder Society–Unified Parkinson’s Disease Rating Scale, MMSE = Mini-Mental State Examination, MoCA = Montreal Cognitive Assessment, MRI = magnetic resonance imaging, SARA = Scale for the Assessment and Rating of Ataxia, SWI = susceptibility-weighted imaging.

## 8. Discussion

The *ELOVL4* variant identified in the present patient, NM_022726.4:c.766_767delinsG (p.Ile256AlafsTer28), represents, to the best of our knowledge, the first frameshift variant reported in a patient with an SCA34-like phenotype. Published SCA34-associated *ELOVL4* variants have been predominantly missense changes,^[1,9–15]^ and their reported genotypic and phenotypic characteristics are summarized in Appendix S1, Supplemental Digital Content 1. In the literature review by Nishide et al, 60 reported patients with SCA34 had a mean age at onset of 31.85 years, and the most common manifestations were gait ataxia, limb ataxia, dysarthria, and nystagmus; cerebellar atrophy was reported in all patients with available MRI data, and the HCBS was observed in a subset of cases.^[15]^ Although the available evidence is insufficient to establish causality, the combination of a previously unreported protein-truncating variant and a clinically compatible phenotype supports its consideration as a candidate variant warranting further investigation.

When discussing mutation-specific severity, however, it is essential to distinguish among the different *ELOVL4*-related disorders rather than comparing all reported *ELOVL4* phenotypes as a single entity. The rapidly progressive childhood case carrying c.503T>C (p.Leu168Ser) was a heterozygous, autosomal dominant SCA34 case with retinal dysfunction; in that report, gait deterioration began at approximately 3.5 years of age, loss of independent ambulation occurred by 4.5 years of age, and death occurred within the first decade of life.^[9]^ By contrast, the Italian cases cited in our original discussion belonged to autosomal recessive *ELOVL4*-related neuro-ichthyosis caused by biallelic variants and were characterized by congenital ichthyosis with severe neurological impairment, rather than autosomal dominant SCA34.^[7]^ Likewise, autosomal dominant Stargardt-like macular dystrophy associated with *ELOVL4* represents another distinct phenotype centered on macular degeneration rather than cerebellar ataxia.^[4,5]^ Therefore, direct comparisons of age at onset, progression rate, and extracerebellar manifestations across dominant SCA34, recessive neuro-ichthyosis, and dominant macular dystrophy should be made with caution.

Clinical heterogeneity is also evident within dominant SCA34 itself. Cutaneous involvement is variably expressed across reported families and variants, and the available literature suggests incomplete penetrance and variable expressivity rather than a simple one-variant/one-phenotype relationship.^[10–15]^ This variability may help explain why some patients develop erythrokeratoderma whereas others present predominantly with cerebellar, retinal, cognitive, or parkinsonian features.

Available mechanistic data further support variant-specific biological effects. Neuropathological examination of a patient with p.Trp246Gly showed pontocerebellar atrophy, vacuolar degeneration of the white matter, and widespread oligodendroglial pathology.^[16]^ In another study, SCA34 associated with p.Leu168Phe showed abnormal neuronal lipid accumulation with lysosomal storage disease-like features,^[17]^ while fibroblast studies demonstrated abnormal subcellular localization of mutant *ELOVL4* protein.^[13]^ In addition, an *ELOVL4* p.Trp246Gly rat model showed synaptic transmission defects in the cerebellum, supporting cerebellar circuit dysfunction as one contributor to disease expression.^[18]^

In the present case, the clinical picture was notable for cerebellar ataxia, parkinsonism, marked cognitive impairment, and autonomic dysfunction. Compared with the family reported by Beaudin et al, in which the mean MoCA score was 18, our patient’s MoCA score of 8 suggests more severe cognitive involvement.^[13]^ However, because this is a single case without segregation analysis, Sanger confirmation, or functional validation of the identified variant, it would be premature to conclude that frameshift variants necessarily produce a more severe neurological phenotype than missense variants. A more conservative interpretation is that this case broadens the observed clinical spectrum associated with *ELOVL4*-related ataxia. The cognitive impairment in this patient may be compatible with cerebellar cognitive-affective involvement, a concept supported by prior SCA34 literature and by broader work on cerebellar contributions to cognition.^[19–21]^

The reduced visibility of the dorsolateral nigral hyperintensity (“swallow-tail” sign) on SWI in this patient is noteworthy, but its interpretation should remain cautious. Nigrosome-1 loss on susceptibility-based MRI has been reported in parkinsonian disorders and is not specific to a particular genetic ataxia.^[22,23]^ In the absence of dopamine transporter imaging, neuromelanin-sensitive MRI, longitudinal imaging, or neuropathological confirmation, any relationship between this qualitative SWI finding and the *ELOVL4* variant remains hypothetical. At present, this imaging observation is best regarded as an ancillary finding that may suggest possible nigrostriatal involvement, rather than evidence of an established lipid-metabolism mechanism.

Overall, this case expands the clinical spectrum of *ELOVL4*-associated ataxia by describing a previously unreported frameshift variant in a patient with cerebellar ataxia, parkinsonism, severe cognitive impairment, and autonomic dysfunction. Because SCA34 can phenotypically overlap with MSA of the cerebellar type, genetic testing remains essential for distinguishing hereditary ataxias from atypical degenerative syndromes.^[10,15]^ Further case accumulation and functional studies will be required to determine whether this variant defines a reproducible genotype–phenotype association.

## 9. Limitations

Several limitations should be considered. First, although disease severity was quantified during hospitalization using the MDS-UPDRS Part III and SARA, no repeat scale assessments were obtained during follow-up. Because the patient declined further in-person evaluation owing to financial difficulties and travel distance, follow-up was conducted primarily by telephone and video consultation. Accordingly, the reported improvement in tremor, gait, and increased muscle tone was based mainly on the patient’s account and could not be confirmed by standardized neurological examination. In addition, although levodopa/benserazide and piribedil were prescribed during hospitalization and reportedly continued for several months, the exact duration of therapy, subsequent dose changes, adherence, and post-discharge medication regimen remained uncertain.

Second, the identified *ELOVL4* frameshift variant was not independently confirmed by Sanger sequencing. Family members were not available for genetic testing, and segregation analysis, therefore, could not be performed. It was consequently not possible to determine whether the variant occurred de novo or segregated with the clinical phenotype. In the absence of independent molecular confirmation, family segregation data, and functional studies, the pathogenic role of this variant cannot be established definitively from a single case. Further studies incorporating Sanger confirmation, family-based analysis, functional characterization, and longitudinal standardized clinical assessments are needed.

## 10. Conclusion

This case describes a previously unreported *ELOVL4* frameshift variant in a patient with cerebellar ataxia, parkinsonism, severe cognitive impairment, and autonomic dysfunction. The predicted protein-truncating effect of the variant and the patient’s phenotypic overlap with *ELOVL4*-related ataxia suggest that the variant may be clinically relevant and may extend the recognized clinical and molecular spectrum of *ELOVL4*-associated ataxia. However, additional cases, independent molecular confirmation, segregation analysis, and functional studies are required to establish a definitive genotype–phenotype relationship.

## 11. Patient’s perspective

Patient and family member’s account 1 year later, recorded and translated by the attending physician:

“I have had difficulty maintaining attention since childhood. I was easily distracted in class, often drowsy, and generally lagged behind others in both academics and physical activities. As a result, I dropped out of school early and did not even finish middle school. In the beginning, I could still take care of myself and managed to find a job to support myself.

However, in my twenties, things started to get worse. I became excessively sleepy and slow to react to everything around me. Speaking became difficult, and sometimes people could not understand what I was saying and asked me to repeat myself several times. I also began choking on water occasionally. Over time, I felt my muscles becoming increasingly stiff, which made my neck constantly feel tight and sore. My legs, especially around the buttocks, thighs, and inner calves, often ached. I also started walking much more slowly, frequently losing my balance and falling.

At night, the tremors in my hands and feet became quite severe. In recent years, my constipation has worsened significantly, often accompanied by abdominal pain. I had visited many hospitals before and even underwent MRI scans, but no clear diagnosis was made. They told me I had “cognitive impairment.”

After receiving treatment at your hospital and taking the prescribed medications, my walking and hand tremors have improved somewhat. However, I still feel that my condition is gradually worsening. People have an even harder time understanding my speech now, and the constipation has become severe – sometimes I go 5 days without a bowel movement, accompanied by intense abdominal pain.”

## Acknowledgments

We thank the patient and his family for their cooperation. We also acknowledge the colleagues in the Department of Neurology who assisted with the genetic interpretation and imaging review. No individual is identified by name in this section.

During the preparation of this manuscript, the authors used ChatGPT (OpenAI) to assist with English-language editing and formatting. The authors reviewed and revised all generated text and take full responsibility for the accuracy, integrity, and content of the manuscript.

## Author contributions

**Data curation:** Jingtong Zhou, Xiaohui Wen.

**Investigation:** Xiaohui Wen.

**Writing – original draft:** Jingtong Zhou.

**Writing – review & editing:** Zhijun Lin.


