# Supplementary material for: A previously unreported ELOVL4 frameshift variant in a patient with early severe cognitive decline, parkinsonism, and cerebellar ataxia: A case report
Source: Medicine (Baltimore). 2026 Sep 25;105(39):e50787. doi: 10.1097/MD.0000000000050787 (PMC13619287; doi:10.1097/MD.0000000000050787)
Supplement: Supplementary file 1 [file medi-105-e50787-s001.doc]

# Supplemental Digital Content 1

**Appendix 1. Reported ELOVL4 Variants and Associated Clinical Phenotypes**

This appendix summarizes published ELOVL4 variants discussed in the manuscript and selected canonical variants associated with the three major ELOVL4-related disease categories. Clinical features are reported as described in the cited publications. NR indicates not reported. Because published case numbers, clinical assessment methods, and variant classifications differ across reports, direct quantitative comparisons should be interpreted cautiously.

**Part A. Heterozygous ELOVL4 variants associated with autosomal dominant spinocerebellar ataxia type 34**

| **HGVS coding variant** | **Predicted protein change** | **Allelic state / inheritance** | **Reported disease category** | **Reported patients or families** | **Age at onset** | **Principal clinical features** | **Skin and retinal findings** | **Neuroimaging / functional evidence** | **Source** |
| --- | --- | --- | --- | --- | --- | --- | --- | --- | --- |
| c.503T>C | p.(Leu168Ser) | Heterozygous; autosomal dominant | SCA34 with retinal dysfunction | 1 patient | Early childhood; gait deterioration at approximately 3.5 years | Rapidly progressive cerebellar ataxia, developmental impairment, loss of independent ambulation by 4.5 years | No erythrokeratoderma reported; retinal dysfunction present | Progressive cerebellar and cerebral atrophy; functional studies showed impaired very-long-chain fatty-acid synthesis | Gyening et al., 2023 |
| c.504G>C | p.(Leu168Phe) | Heterozygous; autosomal dominant | SCA34 | Multigenerational family and additional reported cases | Usually adult onset | Progressive gait and limb ataxia, dysarthria, pyramidal signs; cognitive impairment reported in some affected individuals | Erythrokeratoderma variably present; retinal involvement not a consistent feature | Cerebellar atrophy; abnormal ELOVL4 subcellular localization in fibroblasts; neuronal lipid-storage-like pathology reported | Beaudin et al., 2020; Ellezam et al., 2023 |
| c.512T>C | p.(Ile171Thr) | Heterozygous; autosomal dominant | SCA34 with retinitis pigmentosa | 1 family | Adult onset | Spinocerebellar ataxia with variable extracerebellar manifestations | Retinitis pigmentosa reported in affected family members; skin findings variable or not consistently reported | Cerebellar atrophy | Xiao et al., 2019 |
| c.539A>C | p.(Gln180Pro) | Heterozygous; autosomal dominant | SCA34 with erythrokeratoderma | 1 reported family/case series | Adult onset | Progressive cerebellar ataxia and dysarthria | Erythrokeratoderma present; retinal disease not emphasized | Cerebellar atrophy | Bourassa et al., 2015 |
| c.698C>T | p.(Thr233Met) | Heterozygous; autosomal dominant | SCA34 | Reported in several unrelated families | Variable, generally adult onset | Progressive gait and limb ataxia, dysarthria, nystagmus; cognitive and pyramidal features variably reported | Cutaneous involvement variably present; retinal disease uncommon | Cerebellar atrophy consistently reported; hot cross bun sign present in a subset | Ozaki et al., 2019; Bourque et al., 2018; Nishide et al., 2024 |
| c.736T>G | p.(Trp246Gly) | Heterozygous; autosomal dominant | SCA34 | Reported family and experimental models | Adult onset | Progressive cerebellar ataxia and dysarthria | Erythrokeratoderma absent in the original clinical report; retinal functional abnormalities demonstrated in a rat model | Cerebellar and pontine atrophy; hot cross bun sign; vacuolar white-matter degeneration and oligodendroglial pathology; synaptic transmission defects in rat models | Ozaki et al., 2015, 2021; Nagaraja et al., 2023 |
| c.766_767delinsG | p.(Ile256AlafsTer28) | Heterozygous; inheritance undetermined | SCA34-like phenotype (present case) | 1 patient | Symptoms became clinically prominent in early adulthood | Cerebellar ataxia, parkinsonism, severe cognitive impairment, dysarthria, and autonomic dysfunction | No cutaneous manifestations identified; formal retinal phenotyping was limited | Diffuse cerebral and marked cerebellar atrophy; reduced visibility of the dorsolateral nigral hyperintensity on susceptibility-weighted imaging; no functional validation | Present case; variant classified as a variant of uncertain significance in the clinical report |

**Part B. Biallelic ELOVL4 variants associated with autosomal recessive neuro-ichthyosis**

| **HGVS coding variant** | **Predicted protein change** | **Allelic state / inheritance** | **Reported disease category** | **Reported patients or families** | **Age at onset** | **Principal clinical features** | **Skin and retinal findings** | **Neuroimaging / functional evidence** | **Source** |
| --- | --- | --- | --- | --- | --- | --- | --- | --- | --- |
| c.646C>T | p.(Arg216Ter) | Homozygous; autosomal recessive | Ichthyosis, spastic quadriplegia, and impaired intellectual development | Affected individuals from a consanguineous family | Infancy | Severe developmental impairment, seizures, spasticity, and quadriplegia | Congenital ichthyosis; ocular findings variably reported | Severe neurologic involvement; no SCA34 phenotype | Aldahmesh et al., 2011 |
| c.78C>G | p.(Tyr26Ter) | Homozygous; autosomal recessive | ELOVL4-related neuro-ichthyosis | Affected members of a consanguineous family | Congenital / childhood | Variable neurologic involvement; intellectual disability and spasticity may occur | Ichthyosis; macular vascular changes reported in affected individuals | Neurologic expression was variable within the family | Mir et al., 2014 |
| Multiple biallelic variants, including an exon 1 deletion and homozygous missense variants | Variant-specific | Homozygous; autosomal recessive | ELOVL4-related neuro-ichthyosis | 4 additional unrelated families | Congenital / infancy | Global developmental delay, epilepsy, axial hypotonia, peripheral hypertonia, feeding difficulties, and failure to thrive were variably present | Ichthyosis was present in all reported subjects; ocular abnormalities were variable | Neuroimaging findings varied; biochemical studies supported altered very-long-chain fatty-acid metabolism in selected cases | Alabdulrazzaq et al., 2023 |

**Part C. Heterozygous ELOVL4 variants associated with autosomal dominant Stargardt-like macular dystrophy type 3**

| **HGVS coding variant** | **Predicted protein change** | **Allelic state / inheritance** | **Reported disease category** | **Reported patients or families** | **Age at onset** | **Principal clinical features** | **Skin and retinal findings** | **Neuroimaging / functional evidence** | **Source** |
| --- | --- | --- | --- | --- | --- | --- | --- | --- | --- |
| c.790_794delAACTT (historical numbering also reported as 797_801delAACTT) | Frameshift with C-terminal truncation | Heterozygous; autosomal dominant | Stargardt-like macular dystrophy type 3 | Multiple affected families | Usually first to third decade | Progressive central visual loss without a primary cerebellar syndrome | No characteristic skin disease; macular atrophy and fundus flecks | Loss of the C-terminal endoplasmic-reticulum retention signal; retinal degeneration demonstrated in animal models | Zhang et al., 2001; Vasireddy et al., 2009 |
| c.789delT and c.794delT (complex allele) | Frameshift with C-terminal truncation | Heterozygous; autosomal dominant | Stargardt-like macular dystrophy type 3 | 1 large family | Juvenile / young adult | Progressive macular degeneration without reported cerebellar ataxia | No characteristic skin disease; retinal degeneration | Predicted loss of the C-terminal endoplasmic-reticulum retention signal | Bernstein et al., 2001; reviewed by Vasireddy et al., 2010 |
| c.810C>G | p.(Tyr270Ter) | Heterozygous; autosomal dominant | Stargardt-like macular dystrophy type 3 | Reported in a Belgian family and additional patients | Usually adolescence or young adulthood | Isolated progressive central visual loss; no primary cerebellar syndrome | No characteristic skin disease; macular degeneration | Truncated protein with abnormal cytoplasmic aggregation and loss of normal endoplasmic-reticulum localization | Maugeri et al., 2004; ClinVar RCV000005228 |

**Abbreviations:** ELOVL4 = elongation of very long-chain fatty acids protein 4; NR = not reported; SCA34 = spinocerebellar ataxia type 34.

**Notes:** HGVS descriptions are presented using the transcript nomenclature reported in the cited publications. Historical nucleotide numbering for early Stargardt-like macular dystrophy reports may differ from current MANE transcript numbering. The present case variant was identified by whole-exome sequencing but was not independently confirmed by Sanger sequencing, and family segregation analysis was unavailable.
